# Comparison of survival benefit and safety between surgery following conversion therapy versus surgery alone in patients with surgically resectable hepatocellular carcinoma at CNLC IIb/IIIa stage: a propensity score matching study

**DOI:** 10.1097/JS9.0000000000001193

**Published:** 2024-02-14

**Authors:** Zifeng Ma, Zhiyu Xiao, Pengfei yin, Kai Wen, Weidong Wang, Yongcong Yan, Zijian Lin, Zonglin Li, Haikuo Wang, Jianlong Zhang, Kai Mao

**Affiliations:** a Department of Hepatobiliary Surgery, Sun Yat-Sen Memorial Hospital, Sun Yat-Sen University, Guangzhou; b Department of Hepatopancreatobiliary Surgery, Chongqing General Hospital, Chongqing; People’s Republic of China

**Keywords:** conversion therapy, hepatectomy, hepatocellular carcinoma, propensity score matching, recurrence-free survival, textbook outcome in liver surgery

## Abstract

**Objective:**

The objective of this study is to evaluate and compare the survival benefit and safety of surgery following conversion therapy versus surgery alone in patients diagnosed with surgically resectable hepatocellular carcinoma (HCC) at China Liver Cancer Staging (CNLC) IIb/IIIa stage.

**Methods:**

A total of 95 patients diagnosed with surgically resectable CNLC IIb/IIIa HCC were retrospectively enrolled in our study from November 2018 to December 2022. Among them, 30 patients underwent conversion therapy followed by hepatectomy, while the remaining 65 received surgery alone. The primary endpoint was recurrence-free survival (RFS). Propensity score matching was employed to minimize bias in the retrospective analysis.

**Results:**

Compared to the surgery alone group, the conversion therapy group demonstrated a significantly prolonged median RFS (17.1 vs. 7.0 months; *P*=0.014), a reduced incidence of microvascular invasion (MVI, 23.3 vs. 81.5%; *P*<0.001), and a comparable rate of achieving Textbook Outcome in Liver Surgery (TOLS, 83.3 vs. 76.9%; *P*=0.476). Multivariate analysis indicated that conversion therapy was independently associated with improved RFS after hepatectomy (HR=0.511, *P*=0.027). The same conclusions were obtained after propensity score matching.

**Conclusions:**

The findings of our study offer preliminary evidence that preoperative conversion therapy significantly prolongs RFS in patients with surgically resectable HCC at CNLC IIb/IIIa stage. Furthermore, combining conversion therapy and hepatectomy represents a relatively safe treatment strategy.

## Background

Highlights

- Conversion therapy significantly improved the recurrence-free survival in China Liver Cancer Staging IIb/IIIa hepatocellular carcinoma patients.
- Conversion therapy reduced the risk of hepatocellular carcinoma recurrence by 51.1%.
- Patients treated by conversion therapy reduced their incidence of postoperative microvascular invasion.
- 83.4% patients in the conversion therapy group achieved Textbook Outcome in Liver Surgery.
- Propensity score matching was employed to mitigate potential biases in this research.

The global prevalence of liver cancer ranks it as the sixth most common malignancy, and it stands as the third leading cause of cancer-related mortality, with 905 677 newly diagnosed cases and 830 180 deaths occurring in 2020^1^. Hepatocellular carcinoma (HCC) accounts for over 90% of primary liver cancer cases^2^. Approximately 25–70% of HCC patients receive an advanced-stage diagnosis, which presents limited therapeutic options and an incurable prognosis^3^.

According to China’s healthcare systems and practice experience, the China Liver Cancer Staging (CNLC) system is recommended for HCC patients with stage IIb characterized by the presence of ≥4 nodules and stage IIIa characterized by blood vessel invasion^4^. However, the efficacy of hepatic resection in these patients remains controversial due to the high risk of postoperative recurrence. Clinical guidelines from the National Comprehensive Cancer Network (NCCN) and the Barcelona Cancer Liver Clinic (BCLC) staging system recommend nonsurgical therapies based on systemic or local treatment for such patients^5,6^. Currently, in real-world clinical practice, selected patients with intermediate-advanced HCC who fulfill specific criteria may still be considered for surgical intervention and can achieve negative surgical margins^4,7,8^. Recent attention has been focused on exploring innovative approaches to comprehensive management of intermediate-advanced HCC.

Conversion therapy refers to the process of converting an unresectable HCC into a resectable one, with the aim of enhancing surgical eligibility and prognosis. Unresectable HCC can be categorized into two distinct groups. The first group consists of patients who are considered unsuitable for surgery due to their overall health condition, impaired liver function, and inadequate residual liver volume (surgically unresectable). The second group encompasses technically resectable HCC cases where surgical removal does not provide superior efficacy compared to nonsurgical treatments (oncologically/biologically unresectable). For surgically resectable but oncologically unresectable CNLC IIb/IIIa HCC patients, downstaging conversion to surgical resection is more likely to be achieved since only one of the factors rendering it unresectable is present^9^.

The effectiveness of conversion therapy has gradually been demonstrated. Patients who undergo surgery after conversion therapy can achieve a postoperative 5-year survival rate ranging from 50 to 60%, which is comparable to the prognosis of early-stage HCC patients undergoing surgical resection^9^. Prior to 2018, the lack of established conversion protocols hindered breakthroughs in conversion therapy due to slow advancements in systemic therapy. However, recent years have witnessed significant progress in conversion therapy, thanks to successful targeted therapy and immunotherapy as well as the synergistic effects of combination therapies. Notably, patients with unresectable HCC have achieved remarkable downstaging and improved survival benefits through conversion therapy. The combination of lenvatinib with toripalimab and hepatic arterial infusion chemotherapy (HAIC) demonstrated a significant improvement in the objective response rate (ORR) compared to lenvatinib monotherapy, with rates of 67.6 versus 16.3%, respectively. Additionally, this combination therapy resulted in an extended median progression-free survival of 11.1 months compared to 5.1 months for lenvatinib monotherapy^10^. Similarly positive outcomes were observed in a prospective phase II trial, where the ORR was found to be 66.7%^11^.

In recent years, investigators have applied various conversion therapies to patients with surgically resectable CNLC IIb/IIIa HCC and achieved promising results^12–15^. However, most previous studies have not compared the efficacy and perioperative outcomes of surgery after conversion therapy with those of existing treatment strategies such as surgery alone. Insufficient evidence exists to support the application of conversion therapy in these patients, while the specific mechanisms by which it improves surgical outcomes remain unspecified. This study aimed to compare the survival benefit and safety of surgery alone versus surgery following conversion therapy in patients with surgically resectable HCC at CNLC IIb/IIIa stage. Additionally, PSM was employed to mitigate potential biases inherent in this retrospective research.

## Methods

### Study design and patients

**Figure 1:**
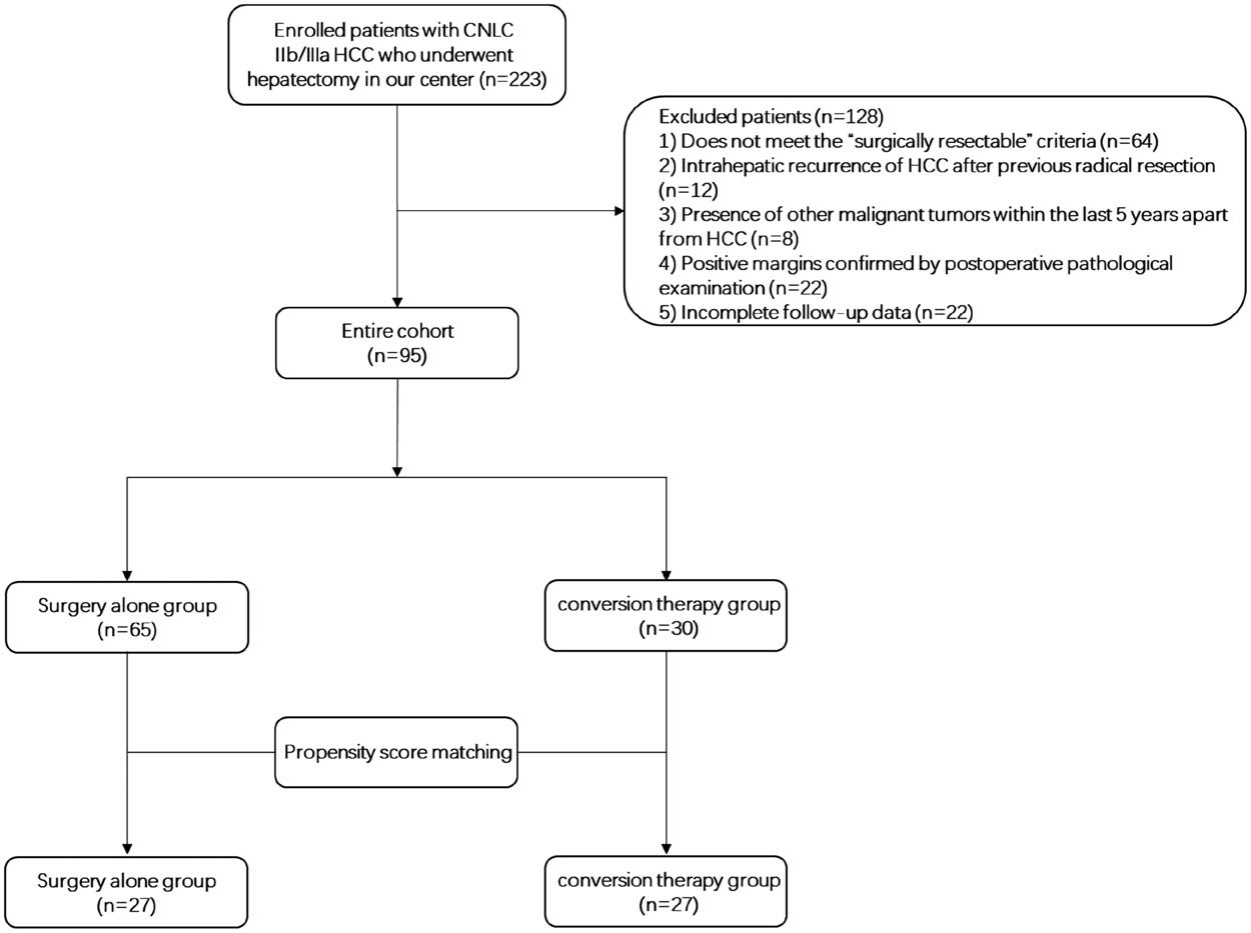
Flowchart of this study.

A total of 223 patients with CNLC IIb/IIIa HCC who underwent hepatectomy at our center between November 2018 and December 2022 were reviewed in this study. The inclusion criteria were as follows: 1) age ≥18 years, 2) confirmation of HCC diagnosis through postoperative pathological examination, 3) CNLC IIb/IIIa stage, 4) meeting the criteria for ‘surgically resectable’, 5) absence of extrahepatic lesions or contraindications to surgery such as impaired vital organ functions, 6) no prior local or systemic treatment received for HCC. The exclusion criteria were presented in Figure 1. The criteria for ‘surgically resectable’ utilized in this study were formulated by a multidisciplinary team (MDT) and comprised of six factors evaluating the surgical resectability of HCC, including: 1) expectation for complete tumor resection (R0 resection), 2) preservation of at least two contiguous liver segments with adequate biliary drainage, inflow and outflow of blood vessels, 3) Eastern Cooperative Oncology Group Performance Status (ECOG PS) score of 0–1, 4) Child–Pugh class A or B, 5) indocyanine green retention rate at 15 min (ICG-R15) <30%, 6) ability to maintain sufficient residual liver volume (residual liver-to-standard liver volume ratio >30% in healthy liver or >40% in liver with chronic disease, parenchymal injury or cirrhosis). A total of 128 instances with invalid data were excluded from analysis, resulting in a final cohort size of 95 cases. Among them, 65 cases underwent hepatectomy alone, while 30 received surgical resection after undergoing preoperative conversion therapy (Fig. 1). After applying PSM, a cohort of patients with similar demographic and clinical characteristics was established. A one-to-one matching was conducted between patients undergoing direct hepatectomy and those in the conversion therapy group. The study protocol received approval from the Ethics Committee on 21 June 2023, and it was registered in the Chinese Clinical Trial Registry. Our research adhered to the strengthening the reporting of cohort, cross-sectional, and case–control studies in surgery (STROCSS) criteria for reporting^16^ (Supplemental Digital Content 1, http://links.lww.com/JS9/B938).

### Conversion therapy and treatment response

The conversion treatment plan was individually developed by a MDT, taking into account the tumor condition and liver function. Common conversion options include systemic therapy, transarterial chemoembolization (TACE), HAIC, transarterial radioembolization, and combination treatments. In addition to considering the antitumor effect of these regimens, safety and accessibility must also be taken into consideration. Adequate attention should be paid to patient responses during conversion therapy. The timing of surgery was determined by the MDT based on objective evaluation results of patients. Tumor response was assessed according to modified Response Evaluation Criteria in Solid Tumors (mRECIST) criteria^17^. ORR was calculated as the sum of complete response (CR) rate and partial response (PR) rate. Major pathological response (MPR) was identified as a reduction in remaining tumor cells in the tumor bed to ≤10%, while pathological complete response (pCR) indicated an absence of viable tumor cells in resected tissue samples. Imaging and pathology assessments were performed by two professionals specialized in their resive fields. Adverse events (AEs) were evaluated and graded based on Common Terminology Criteria for Adverse Events version 5.0^18^.

### Hepatectomy and complications

Preoperative assessments ensured adequate residual liver volume and absence of absolute surgical contraindications. Patients in the conversion therapy group experienced no significant adverse effects during treatment and were required to discontinue medication intake according to tyrosine kinase inhibitor and programmed cell death 1 (PD-1) inhibitor guidelines prior to surgery. Hepatectomy techniques, including laparotomy or laparoscopy, were selected. The liver parenchyma was precisely incised using an ultrasound scalpel at a minimum distance of 1 cm from the tumor margin. Careful separation or suturing of exposed hepatic veins and bile ducts was performed. Bile ducts were ligated and sutured with Prolene sutures to minimize the risk of bile leakage.

The classification of postoperative complications was based on the Clavien–Dindo grading system^19^. The diagnosis and severity assessment of liver failure following hepatic resection relied upon the definition and grading criteria proposed by the International Study Group of Liver Surgery^20^. Surgical safety evaluation was conducted through the assessment of textbook outcomes (TO). In our study, a consensus-based definition of TO was established by a multinational panel comprising hepatobiliary and pancreatic experts. This expert panel agreed upon five surgery-related indicators for TOLS^21^.

### Follow-up

The decision to administer adjuvant therapy following hepatectomy was based on the patient’s pathological assessment. Patient follow-up was conducted via telephone or outpatient until July 2023 to document survival status and tumor recurrence. Patients were monitored at intervals of every 6 months for a period of 2 years postsurgery, followed by annual monitoring thereafter. RFS was defined as the duration between the surgery date and either tumor recurrence or death from any cause. Overall survival (OS) was defined as the time interval between the surgery date and patient death.

### Statistical analysis

Statistical comparisons between groups were conducted using Student’s *t*-test, Mann–Whitney *U* test, *χ* ^2^ test, and Fisher’s exact test. Survival curves were generated using the Kaplan–Meier method. In univariate analyses, significant factors associated with survival (*P*<0.1) and variables clinically considered to be closely related to survival were included in the Cox proportional hazards model, where the hazard ratios described the differences in survival data between groups. A two-sided *P*-value of <0.05 indicated statistical significance. PSM was performed to control for confounders in both groups by including age, sex, ECOG PS score, Child–Pugh class, history of hepatitis, HBV-DNA level, alpha fetoprotein (AFP) level, CNLC stage, tumor number, maximum tumor diameter, portal vein tumor thrombosis, and cirrhosis as covariates with a caliper size of 0.02. Data analyses was performed using SPSS 26.0, MedCalc 20.022, and GraphPad Prism 9.0.

## Results

### Baseline characteristics

Our study retrospectively analyzed a total of 223 patients with CNLC IIb/IIIa HCC who underwent liver resection at our center between November 2018 and December 2022. After applying rigorous inclusion and exclusion criteria, we established a cohort comprising 95 eligible patients. Subsequently, this cohort was divided into two groups: the conversion therapy group (30 patients), who received conversion therapy prior to liver resection, and the surgery alone group (65 patients), who underwent liver resection without any conversion therapy. A comparison of baseline characteristics between the two groups is presented in Table 1. Notably, before PSM, patients in the conversion therapy group exhibited elevated prothrombin time (*P*=0.026), an increased prothrombin time-international normalized ratio (PT-INR) values (*P*=0.041), and a higher number of lesions (*P*=0.049). After performing 1:1 matching, we obtained a final cohort consisting of 27 pairs of matched patients with no discernible differences detected in their baseline characteristics (Table 1).

**Table 1:** Comparison of the baseline characteristics between the conversion therapy group and surgery alone group.

	Before matching			After matching		
Variables	Surgery alone (*n*=65)	Conversion therapy (*n*=30)	*P*	Surgery alone (*n*=27)	Conversion therapy (*n*=27)	*P*
Age	52.3±11.7	53.1±9.7	0.722	53.4±11.2	53.4±9.7	0.990
Sex			0.192			1.000
Female	10 (15.4%)	8 (26.7%)		7 (25.9%)	7 (25.9%)	
Male	55 (84.6%)	22 (73.3%)		20 (74.1%)	20 (74.1%)	
Chronic disease			0.216			0.669
No	54 (83.1%)	28 (93.3%)		23 (85.2%)	25 (92.6%)	
Yes	11 (16.9%)	2 (6.7%)		4 (14.8%)	2 (7.4%)	
ECOG PS			0.981			0.551
0	24 (36.9%)	11 (36.7%)		7 (25.9%)	9 (33.3%)	
1	41 (63.1%)	19 (63.3%)		20 (74.1%)	18 (66.7%)	
Hemoglobin	142.3±23.9	135.4±14.0	0.083	141.9±22.2	136.1±14.2	0.254
Platelet	241.4±92.8	236.8±91.0	0.825	269.0±84.7	238.0±92.7	0.205
PT	12.0 [11.2,12.5]	12.4 [11.9,13.1]	**0.026**	12.2±1.1	12.5±0.9	0.449
INR	1.0 [1.0,1.1]	1.1 [1.0,1.1]	**0.041**	1.1±0.1	1.1±0.1	0.723
ALT	39.3 [22.0,47.0]	41.7 [19.5,50.5]	0.936	33.9 [21.0,43.0]	39.5 [20.8,44.8]	0.931
AST	59.5 [30.0,65.0]	62.0 [35.5,79.0]	0.148	70.4 [34.0,94.0]	59.7 [35.8,75.5]	0.653
TBil	17.2 [12.0,18.3]	22.7 [11.9,21.1]	0.920	16.2 [13.8,18.2]	24.0 [12.1,22.6]	0.616
GGT	150.1 [47.0,202.0]	142.6 [79.5,185.0]	0.251	160.0 [40.0,247.0]	139.4 [80.8,177.3]	0.586
ALP	122.5 [83.0,159.0]	140.7 [100.0,152.5]	0.133	130.8 [69.0,172.0]	138.3 [100.5,149.8]	0.382
Albumin	36.6±5.7	37.7±4.6	0.351	35.8±5.7	38.3±4.2	0.074
Cholinesterase	6581.7±1939.6	6712.5±2208.8	0.771	6237.9±2026.3	6598.4±1976.8	0.511
			0.581			0.250
Negative	12 (18.5%)	7 (23.3%)		2 (7.4%)	6 (22.2%)	
Positive	53 (81.5%)	23 (76.7%)		25 (92.6%)	21 (77.8%)	
HBV-DNA			0.458			0.783
<104 IU/ml	42 (64.6%)	17 (56.7%)		15 (55.6%)	16 (59.3%)	
≥104 IU/ml	23 (35.4%)	13 (43.3%)		12 (44.4%)	11 (40.7%)	
History of hepatitis			0.715			1.000
No	7 (10.8%)	2 (6.7%)		1 (3.7%)	2 (7.4%)	
Yes	58 (89.2%)	28 (93.3%)		26 (96.3%)	25 (92.6%)	
AFP			0.743			0.413
<400 ng/ml	28 (43.1%)	14 (46.7%)		14 (51.9%)	11 (40.7%)	
≥400 ng/ml	37 (56.9%)	16 (53.3%)		13 (48.1%)	16 (59.3%)	
ICG-R15			0.720			1.000
<10%	59 (90.8%)	26 (86.7%)		23 (85.2%)	23 (85.2%)	
≥10%	6 (9.2%)	4 (13.3%)		4 (14.8%)	4 (14.8%)	
RLV/SLV (%)	60.28 [47.38,71.56]	56.46 [41.08,72.01]	0.321	60.32 [51.63,71.79]	56.54 [43.16,72.79]	0.749
CNLC			0.208			1.000
IIb	12 (18.5%)	9 (30.0%)		7 (25.9%)	7 (25.9%)	
IIIa	53 (81.5%)	21 (70.0%)		20 (74.1%)	20 (74.1%)	
Tumor number	2.2 [1.0,4.0]	3.2 [1.0,5.0]	**0.049**	3.0 [1.0,5.0]	3.0 [1.0,4.3]	0.796
Maximum tumor diameter	9.3±3.4	9.4±3.2	0.942	10.7±3.8	9.5±3.3	0.221
TBS	9.8±3.3	10.1±3.1	0.626	11.2±3.7	10.1±3.2	0.248
PVTT			0.842			0.956
No	24 (36.9%)	13 (43.3%)		11 (40.7%)	11 (40.7%)	
II	18 (27.7%)	7 (23.3%)		4 (14.8%)	6 (22.2%)	
III	22 (33.8%)	7 (23.3%)		11 (40.7%)	7 (25.9%)	
IV	1 (1.5%)	3 (10.0%)		1 (3.7%)	3 (11.1%)	
HVTT			0.351			0.527
No	36 (55.4%)	20 (66.7%)		16 (59.3%)	18 (66.7%)	
II	3 (4.6%)	1 (3.3%)		0 (0.0%)	1 (3.7%)	
III	25 (38.5%)	8 (26.7%)		10 (37.0%)	7 (25.9%)	
IV	1 (1.5%)	1 (3.3%)		1 (3.7%)	1 (3.7%)	
Cirrhosis			0.208			0.535
No	53 (81.5%)	21 (70.0%)		21 (77.8%)	19 (70.4%)	
Yes	12 (18.5%)	9 (30.0%)		6 (22.2%)	8 (29.6%)	
Ascites			1.000			0.484
No	55 (84.6%)	26 (86.7%)		21 (77.8%)	23 (85.2%)	
Mild	10 (15.4%)	4 (13.3%)		6 (22.2%)	4 (14.8%)	
Child–Pugh class			0.738			1.000
A	58 (89.2%)	26 (86.7%)		24 (88.9%)	24 (88.9%)	
B	7 (10.8%)	4 (13.3%)		3 (11.1%)	3 (11.1%)	
ASA			0.368			0.402
II	24 (36.9%)	14 (46.7%)		9 (33.3%)	12 (44.4%)	
III–IV	41 (63.1%)	16 (53.3%)		18 (66.7%)	15 (55.6%)	

Bold values indicate that the value is less than 0.05.

AFP, alpha fetoprotein; ALP, alkaline phosphatase; ALT, alanine aminotransferase; ASA, American Society of Anesthesiologists physical status classification system; AST, aspartate aminotransferase; CNLC, The China liver cancer staging system; ECOG PS, Eastern Cooperative Oncology Group Performance Status; GGT, gamma-glutamyl transpeptidase; HBsAg, hepatitis B surface antigen; HVTT, hepatic vein tumor thrombosis; ICG-R15, indocyanine green retention rate at 15 min; INR, international normalized ratio; PT, prothrombin time; PVTT, portal vein tumor thrombosis; RLV/SLV, residual liver-to-standard liver volume ratio; TBil, total bilirubin; TBS, tumor burden score.

Statistical significance *P* < 0.05 values are in bold.

### RFS and OS

**Figure 2:**
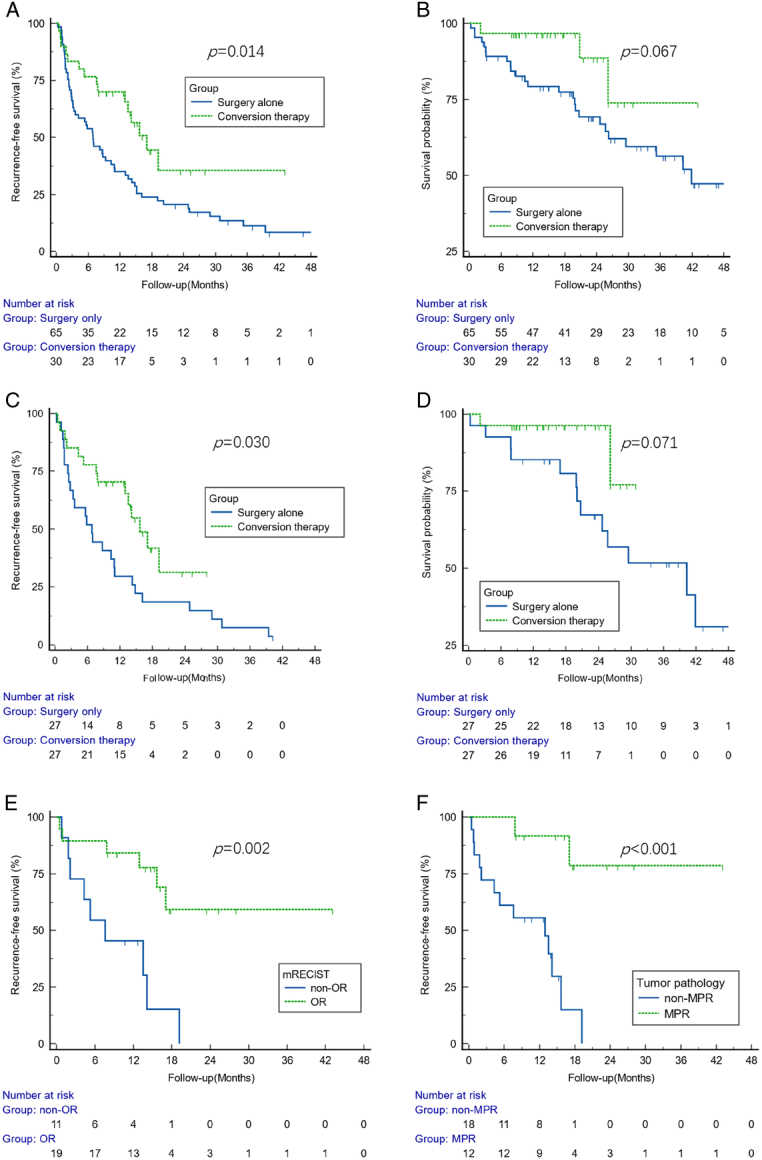
The recurrence-free survival (A) and overall survival (B) curves for the conversion therapy group and the surgery alone group. The recurrence-free survival (C) and overall survival (D) curves for the conversion therapy group and the surgery alone group after propensity matching. (E) The recurrence-free survival curves for the objective response in the conversion therapy group. (F) The recurrence-free survival curves for the major pathological response in the conversion therapy group. MPR, major pathological response; OR, objective response.

The median follow-up duration for patients in the conversion therapy group was 17.6 (12.7–23.4) months, while it extended to 35.1 (23.4–40.2) months in the surgery alone group. During the observation period, tumor recurrence was observed in a total of 14 patients receiving conversion therapy and compared with that of 50 individuals who underwent surgery alone. The RFS rates at months 6, 12, and 24 were higher in the conversion therapy group compared to the surgery alone group: 76.7 vs. 53.8%, 70.0 vs. 35.1%, and 35.6 vs. 20.7%, respectively. A notable disparity was noted when comparing median RFS between these two groups [17.1 (12.9–19.2) vs. 6.9 (3.3–10.3) months; *P*=0.014] (Fig. 2A). Multivariate Cox regression analysis revealed that conversion therapy acted as a substantial protective factor for RFS (HR=0.511, 95% CI=0.281–0.927, *P*=0.027) (Table 2). During the observation period, HCC-related deaths were reported in three patients from the conversion therapy group and 25 patients from the surgery alone group. The OS rates at 6, 12, and 24 months for the conversion therapy group versus the surgery alone group were as follows: 96.7 vs. 89.2%, 96.7 vs. 79.3%, and 88.6 vs. 69.3%, respectively. Regarding the median OS, there was no discernible difference between the two groups. (not reached vs. 41.9 (26.3–41.9) months; *P*=0.067) (Fig. 2B). Similar results were obtained using propensity model analysis (Fig 2C, D). After performing a one-to-one matching analysis, conversion therapy remained a significant protective factor for RFS (HR=0.453, 95% CI=0.211–0.972, *P*=0.042) (Table 2).

**Table 2:** Univariate and multivariate analyses of recurrence-free survival in patients receiving surgery following conversion therapy or surgery alone.

Variables	UV HR (95% CI)	*P*	MV HR (95% CI)	*P*
Before matching				
Age (≤60 vs. >60 years)	1.642 (1.003–2.687)	**0.049**		
Sex (Female vs. Male)	0.731 (0.423–1.264)	0.263		
Chronic disease (No vs. Yes)	1.392 (0.757–2.561)	0.287		
ECOG PS (0 vs. 1)	0.922 (0.572–1.486)	0.738		
ALT (≤40 vs. >40 U/l)	0.699 (0.425–1.150)	0.159		
AST (≤35 vs. >35 U/l)	0.724 (0.443–1.182)	0.197		
HBsAg (Negative vs. Positive)	0.717 (0.404–1.272)	0.255		
HBV-DNA (<10 000 vs. ≥10 000 IU/ml)	1.087 (0.674–1.753)	0.733		
History of hepatitis (No vs. Yes)	0.853 (0.386–1.888)	0.696		
AFP (＜400 vs. ≥400 ng/ml)	0.647 (0.406–1.032)	0.068		
CNLC (IIb vs. IIIa)	0.678 (0.402–1.145)	0.146		
Tumor number (Single vs. Multiple)	1.269 (0.795–2.026)	0.318		
Tumor diameter (≤10 vs. >10 cm)	1.029 (0.644–1.643)	0.905		
PVTT (0–II vs. III–Ⅳ)	0.920 (0.555–1.524)	0.745		
Cirrhosis (No vs. Yes)	1.181 (0.686–2.034)	0.549		
Ascites (No vs. Yes)	0.455 (0.207–1.001)	0.050		
Child–Pugh class (A vs. B)	0.715 (0.325–1.572)	0.404		
Treatment group (Conversion therapy vs. surgery alone)	0.538 (0.329–0.881)	**0.014**	0.511 (0.281–0.927)	**0.027**
After matching				
Age (≤60 vs. >60 years)	1.464 (0.756–2.836)	0.258		
Sex (Female vs. Male)	0.733 (0.373–1.443)	0.369		
Chronic disease (No vs. Yes)	1.470 (0.638–3.389)	0.366		
ECOG PS (0 vs. 1)	0.712 (0.365–1.386)	0.317		
ALT (≤40 vs. >40 U/l)	0.494 (0.237–1.029)	0.060		
AST (≤35 vs. >35 U/l)	0.515 (0.264–1.007)	0.052		
HBsAg (Negative vs. Positive)	0.617 (0.265–1.440)	0.264		
HBV-DNA (<10 000 *vs.* ≥10 000 IU/ml)	0.935 (0.490–1.783)	0.838		
History of hepatitis (No vs. Yes)	1.459 (0.278–7.667)	0.656		
AFP (＜400 vs. ≥400 ng/ml)	0.638 (0.334–1.217)	0.172		
CNLC (IIb vs. IIIa)	0.546 (0.283–1.053)	0.071		
Tumor number (Single vs. Multiple)	1.068 (0.548–2.081)	0.846		
Tumor diameter (≤10 vs. >10 cm)	1.076 (0.573–2.018)	0.820		
PVTT (0–II vs. III–Ⅳ)	0.722 (0.364–1.433)	0.351		
Cirrhosis (No vs. Yes)	1.556 (0.790–3.066)	0.201		
Ascites (No vs. Yes)	0.453 (0.171–1.200)	0.111		
Child–Pugh class (A vs. B)	0.987 (0.347–2.806)	0.980		
Treatment group (Conversion therapy vs. surgery alone)	0.484 (0.251–0.930)	**0.030**	0.453 (0.211–0.972)	**0.042**

Bold values indicate that the value is less than 0.05.

AFP, alpha fetoprotein; ALT, alanine aminotransferase; AST, aspartate aminotransferase; CNLC, The China liver cancer staging system; ECOG PS, Eastern Cooperative Oncology Group Performance Status; HBsAg, hepatitis B surface antigen; PVTT, portal vein tumor thrombosis.

Statistical significance *P* < 0.05 values are in bold.

Subgroup analysis of RFS demonstrated that hepatectomy following conversion therapy yielded greater clinical benefits in specific patient subgroups, including those who were younger (≤60 years), male, had lower levels of alanine aminotransferase (ALT) (≤40 U/l), tested negative for hepatitis B surface antigen (HBsAg), exhibited higher AFP levels (≥400 ng/ml), belonged to Child–Pugh class A with better liver function, had larger tumors (≥10 cm), and were classified as CNLC IIIa. These findings are illustrated in Figure S1 of the Supplemental Digital Content 2, http://links.lww.com/JS9/B939.

### Results of pathological examination

R0 resection was achieved in all cases. Postoperative pathological examination revealed that, compared to the surgery alone group, the tumors resected from the conversion therapy group exhibited smaller diameters (*P*=0.012), higher grades of tumor differentiation (*P*=0.018), and a lower incidence of hepatic capsule invasion (*P*<0.001) as well as MVI (*P*<0.001) (Table 3).

**Table 3:** Comparison of surgical and postoperative characteristics and the results of pathological examination between the conversion therapy group and surgery alone group.

	Before matching			After matching		
Variables	Surgery alone (*n*=65)	Conversion therapy (*n*=30)	*P*	Surgery alone (*n*=27)	Conversion therapy (*n*=27)	*P*
Operative time	267.8 [202.0,330.0]	284.6 [220.0,345.0]	0.494	256.7 [175.0,310.0]	288.9 [223.8,340.0]	0.164
Perioperative blood loss			0.757			0.580
≤400 ml	39 (60.0%)	19 (63.3%)		15 (55.6%)	17 (63.0%)	
>400 ml	26 (40.0%)	11 (36.7%)		12 (44.4%)	10 (37.0%)	
Technique of hepatectomy			0.575			1.000
Laparotomy	35 (53.8%)	18 (60.0%)		15 (55.6%)	15 (55.6%)	
Laparoscopy	30 (46.2%)	12 (40.0%)		12 (44.4%)	12 (44.4%)	
Resection extent			0.981			0.573
Minor	24 (36.9%)	11 (36.7%)		9 (33.3%)	11 (40.7%)	
Major	41 (63.1%)	19 (63.3%)		18 (66.7%)	16 (59.3%)	
Embolectomy			1.000			1.000
No	57 (87.7%)	26 (86.7%)		25 (92.6%)	24 (88.9%)	
Yes	8 (12.3%)	4 (13.3%)		2 (7.4%)	3 (11.1%)	
Lymph node dissection			0.493			0.051
No	56 (86.2%)	28 (93.3%)		22 (81.5%)	27 (100.0%)	
Yes	9 (13.8%)	2 (6.7%)		5 (18.5%)	0 (0.0%)	
Postoperative ICU transfer			0.426			0.610
No	59 (90.8%)	29 (96.7%)		24 (88.9%)	26 (96.3%)	
Yes	6 (9.2%)	1 (3.3%)		3 (11.1%)	1 (3.7%)	
Postoperative blood transfusion			0.747			0.704
No	56 (86.2%)	27 (90.0%)		22 (81.5%)	24 (88.9%)	
Yes	9 (13.8%)	3 (10.0%)		5 (18.5%)	3 (11.1%)	
Postoperative hospitalization duration	10.8 [7.0,12.0]	12.0 [7.0,13.0]	0.606	12.1 [7.0,13.0]	11.7 [7.0,13.0]	0.676
Posthepatectomy liver failure			0.747			1.000
No	56 (86.2%)	27 (90.0%)		24 (88.9%)	25 (92.6%)	
Yes	9 (13.8%)	3 (10.0%)		3 (11.1%)	2 (7.4%)	
Complication			0.615			0.917
No	21 (32.3%)	6 (20.0%)		8 (29.6%)	6 (22.2%)	
I	29 (44.6%)	18 (60.0%)		12 (44.4%)	16 (59.3%)	
II	6 (9.2%)	4 (13.3%)		3 (11.1%)	4 (14.8%)	
III	3 (4.6%)	1 (3.3%)		1 (3.7%)	0 (0.0%)	
IV	5 (7.7%)	1 (3.3%)		2 (7.4%)	1 (3.7%)	
V	1 (1.5%)	0 (0.0%)		1 (3.7%)	0 (0.0%)	
Postoperative adjuvant therapy			0.281			0.761
No	27 (41.5%)	9 (30.0%)		8 (29.6%)	7 (25.9%)	
Yes	38 (58.5%)	21 (70.0%)		19 (70.4%)	20 (74.1%)	
TOLS			0.476			0.484
Not achieved	15 (23.1%)	5 (16.7%)		6 (22.2%)	4 (14.8%)	
Achieved	50 (76.9%)	25 (83.3%)		21 (77.8%)	23 (85.2%)	
Resected tumor diameter	10.0 [7.5,12.0]	8.0 [4.5,11.5]	**0.012**	11.2 [9.0,14.0]	8.4 [4.5,12.3]	**0.014**
Tumor differentiation			**0.018**			0.055
Poor	18 (27.7%)	2 (6.7%)		6 (22.2%)	2 (7.4%)	
Moderate	45 (69.2%)	26 (86.7%)		21 (77.8%)	23 (85.2%)	
Well	2 (3.1%)	2 (6.7%)		0 (0.0%)	2 (7.4%)	
Hepatic capsule invasion			**<0.001**			**<0.001**
No	8 (12.3%)	19 (63.3%)		4 (14.8%)	18 (66.7%)	
Yes	57 (87.7%)	11 (36.7%)		23 (85.2%)	9 (33.3%)	
MVI			**<0.001**			**<0.001**
No	12 (18.5%)	23 (76.7%)		6 (22.2%)	20 (74.1%)	
Yes	53 (81.5%)	7 (23.3%)		21 (77.8%)	7 (25.9%)	
Satellite nodule			0.063			**0.008**
No	42 (64.6%)	25 (83.3%)		14 (51.9%)	23 (85.2%)	
Yes	23 (35.4%)	5 (16.7%)		13 (48.1%)	4 (14.8%)	

Bold values indicate that the value is less than 0.05.

MVI, microvascular invasion; TOLS, Textbook Outcome in Liver Surgery.

Statistical significance *P* < 0.05 values are in bold.

The adjusted pathological characteristics of both groups through PSM analysis are also presented in Table 3. The conversion therapy group demonstrated smaller diameters (*P*=0.014), a lower incidence of hepatic capsule invasion (*P*<0.001) and MVI (*P*<0.001) as well as fewer satellite nodules (*P*=0.008) compared with the surgery alone group (Table 3).

### Surgical and postoperative characteristics

As presented in Table 3, there were no significant differences observed between the two groups in terms of surgical and postoperative characteristics. In the surgery alone group, a total of 89 cases of postoperative complications were observed, with pain (*n*=18), ascites (*n*=12), and fever (*n*=10) being the most common. On the other hand, the conversion therapy group had 53 cases of complications, with pain (*n*=14) and vomiting (*n*=9) being predominant (Table S3, Supplemental Digital Content 2, http://links.lww.com/JS9/B939). Moreover, there was no decrease in the proportion of patients achieving TOLS in the conversion therapy group compared to the surgery alone group (83.4 vs. 76.9%, *P*=0.476, Table 3). No statistically significant difference was observed in the utilization of adjuvant therapy after surgery between the two groups (*P*=0.281). Following PSM analysis, no significant statistical differences was found between the two groups concerning these surgical and postoperative characteristics (Table 3). Unfortunately, a patient from the surgery alone group passed away due to posthepatectomy liver failure and abdominal hemorrhage.

### Treatment responses and adverse effects of conversion therapy

**Figure 3:**
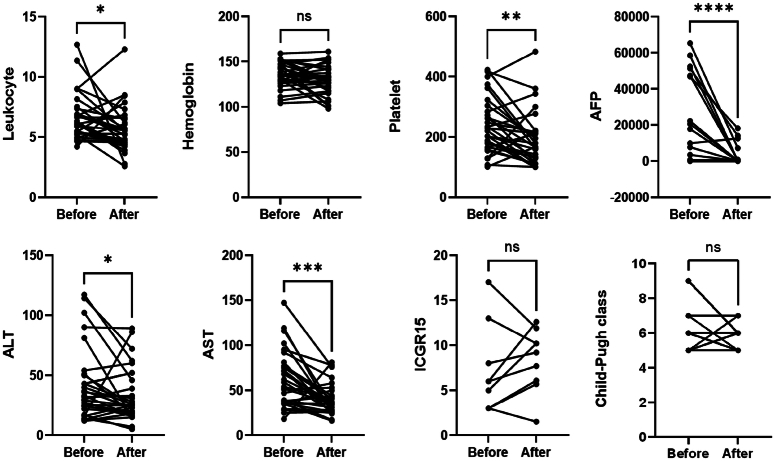
Revisions in blood parameters, AFP levels, and liver function preconversion and postconversion therapy. *P<0.05, **P<0.01, ***P<0.001, ****P<0.0001. AFP, alpha fetoprotein; ALT, alanine aminotransferase; AST, aspartate aminotransferase; ICG-R15, indocyanine green retention rate at 15 min; ns, no significance.

The preoperative conversion protocols for the conversion therapy group comprised PD-1 immune checkpoint inhibitors (ICIs), antiangiogenic agents, and/or local treatment such as TACE or HAIC. The detailed procedures of conversion therapy are provided in Tables S1, S2 (Supplemental Digital Content 2, http://links.lww.com/JS9/B939). Patients in the conversion therapy group underwent a median period of 98 days from the initiation of conversion therapy to surgery. By comparing AFP level before and after conversion therapy, a significant decrease in AFP level was observed (Fig. 3). Following conversion therapy, objective response (OR) was achieved in 19 patients (63.3%), CR in five patients (16.7%). Based on postoperative pathological examination, MPR was achieved in 12 patients (40.0%), with pCR seen in five patients (16.7%) (Table S4, Supplemental Digital Content 2, http://links.lww.com/JS9/B939). Furthermore, survival analysis indicated that patients who achieved MPR exhibited a more favorable prognosis (*P*<0.001), as did those who demonstrated OR (*P*=0.002) (Fig 2E, F). Importantly, all patients who achieved pCR remained free from tumor recurrence during the follow-up period.

Furthermore, we conducted a comparative analysis of liver function and blood parameters before and after conversion therapy. Notably, there was no significant increase in ICG-R15 and the difference in Child–Pugh class was not statistically significant following conversion therapy. Moreover, the ALT levels (*P*=0.038) and AST levels (*P*<0.001) exhibited a significant decrease. However, leukocytes (*P*=0.010) and platelets (*P*<0.001) demonstrated a notable decline following conversion therapy (Fig. 3). Out of the 30 patients who underwent conversion therapy, 28 individuals (90.3%) experienced at least one AE; however, most were categorized as mild in severity. The predominant AEs included anemia in 16 cases (51.6%), elevated AST levels in 12 cases (38.7%), elevated ALT levels in seven cases (22.6%), and thrombocytopenia in seven cases (22.6%), respectively (Table S5, Supplemental Digital Content 2, http://links.lww.com/JS9/B939). Additionally, grade 3 AEs were reported by seven patients (22.6%). No instances of grade 4 or grade 5 AEs were observed (Table S4, Supplemental Digital Content 2, http://links.lww.com/JS9/B939).

## Discussion

Despite the fact that selected patients with surgically resectable CNLC IIb/IIIa HCC still receive surgical intervention in real-world clinical practice^4,7,8^, these individuals exhibit a heightened incidence of early recurrence following hepatectomy and poor long-term survival. Therefore, it is crucial to explore novel treatment options aimed at reducing the risk of postoperative recurrence.

Conversion therapy has emerged as a viable treatment modality for unresectable liver cancer, involving the administration of systemic and local therapies prior to surgery with the aim of reducing tumor size and retracting tumor thrombosis. This approach facilitates the conversion of an initially unresectable HCC into a resectable one, thereby improving survival outcomes for selected patients with intermediate-advanced HCC^9^.

The primary target population for conversion therapy comprises individuals with CNLC IIb/IIIa HCC. Unresectability in HCC can be attributed to both surgical and oncological factors. For patients with surgically resectable but oncologically unresectable CNLC IIb/IIIa HCC, eliminating the unresectable factor is relatively less challenging compared to dealing with two concurrent unresectable factors. Consequently, these patients are more likely to achieve downstaging towards surgical resection. Therefore, conversion therapy can be employed more aggressively in this subgroup of patients aiming at achieving rapid tumor shrinkage and downstaging, ultimately providing an opportunity for radical treatment. Notably, a phase II clinical trial demonstrated that combination therapy utilizing camrelizumab and apatinib in surgically resectable HCC yielded a 1-year RFS rate of 53.85%^22^.

Conversion therapy has a long-standing history, albeit with limited success rates, which has hindered its widespread recognition. Encouragingly, the advent of systemic antitumor regimens such as ‘atezolizumab plus bevacizumab’ and lenvatinib has significantly enhanced the efficacy of conversion therapy. Combination protocols have emerged as the leading therapeutic options in this context. Notably, a study demonstrated that the combination of cabozantinib and navulizumab improved resectability in patients with locally advanced HCC who did not meet traditional resection criteria, thereby extending their long-term RFS^23^. Furthermore, another study revealed that targeted therapy and immunotherapy combined with HAIC resulted in a superior ORR while maintaining manageable toxic side effects^12^. In our conversion protocols, triple combination therapies accounted for 76.7% of cases, yielding an ORR of 63.3%, consistent with previous research findings.

Conversion therapy enhances the survival outcomes of patients with HCC. In our study, the group receiving conversion therapy exhibited a significantly higher 1-year RFS rate compared to the surgery alone group (70.0 vs. 35.1%), which is consistent with previous studies^22,23^. These findings suggested that conversion therapy may provide an additional benefit in terms of RFS for surgically resectable CNLC IIb/IIIa HCC patients. The results from the propensity model further validated the effectiveness of conversion therapy. With respect to OS, our survival analysis indicated a tendency towards prolonged OS associated with conversion therapy. However, due to limited follow-up time, statistical significance in OS has not been achieved between the two groups yet; therefore, additional follow-up is required to confirm whether conversion therapy can extend OS for these patients.

The triple combination therapy has demonstrated promising outcomes in clinical research due to its potential synergistic effect on antitumor mechanisms. Firstly, chemotherapy can modulate the tumor microenvironment to enhance immunological response when combined with ICIs. Chemotherapy agents have the ability to upregulate HLA1 expression in tumor cells and stimulate their secretion of cytokines that promote dendritic cell maturation, ultimately improving T-cell responses and restoring immunosurveillance^24,25^. Additionally, chemotherapy can indirectly activate the immune system by inducing immunogenic cell death in tumor cells, thereby increasing antigenicity^24,26^. Secondly, antiangiogenic agents can normalize and remodel the tumor vasculature, facilitating efficient infiltration of effector immune cells into the tumor microenvironment while alleviating immunosuppression. Conversely, immunotherapy can also induce changes in the vasculature within tumors, creating a positive cycle of immune stimulation and vascular remodeling^27,28^.

The presence of MVI in HCC has consistently been demonstrated as a significant risk factor for early HCC recurrence following hepatectomy^29–32^. Therefore, it is imperative to develop therapeutic strategies aimed at minimizing the occurrence of MVI in order to reduce postoperative recurrence and improve prognosis. In our study, we observed a significantly lower incidence of postoperative MVI in the conversion therapy group compared to the surgery alone group, suggesting that preoperative conversion therapy may effectively reduce the incidence of MVI. Tumor cells acquire an invasive phenotype facilitating MVI through regulation of specific oncogenes and suppressor genes^33^. The reduction of MVI observed in conversion therapy is primarily attributed to the mechanism of systemic therapy and local treatment. Firstly, the synergistic modulation of effector T-cell function and promotion of tumor vessel normalization by antiangiogenic drugs and immunotherapy inhibit tumor cell invasiveness, ultimately leading to a decrease in MVI^34^. Secondly, TACE promotes infiltration of inflammatory cells around the tumor and enhances the integrity of the tumor capsule^35^, which is associated with MVI occurrence^36^. Figure S2 (Supplemental Digital Content 2, http://links.lww.com/JS9/B939) illustrates the potential mechanisms through which conversion therapy reduces MVI and improves patient prognosis. The observed reduction in hepatic capsule invasion and satellite nodules may also involve these aforementioned mechanisms. Additionally, we found that patients who underwent conversion therapy had significantly higher grades of tumor differentiation. We hypothesize that conversion therapy may alter tumor differentiation and biological characteristics. However, due to heterogeneity in our conversion protocols, it remains unclear from our study which specific protocols contributed to improved pathological outcomes. Meanwhile, as preoperative biopsies were lacking, accurate determination of baseline tumor status was not possible; thus, further studies are needed for validation.

All patients underwent curative surgical resection subsequent to conversion therapy, including six patients with progressive disease. Previous studies have demonstrated that even with continued medication, tumor progression occurred within 1.0 to 1.5 years for those who achieved a response to therapy^37^. This suggests that surgical resection may be necessary following conversion therapy. In cases of rapid tumor progression, the MDT developed personalized follow-up strategies, based on the patient’s previous treatments and disease progression patterns, such as modifying the conversion protocols or performing surgery. We posit that prompt surgical intervention subsequent to efficacy assessments of conversion therapy can mitigate surgery timing delays and enhance patient survival, even in instances of tumor progression. However, the optimal timing of surgery following conversion therapy remains a subject of controversy.

To further evaluate surgical quality and surgical safety between the two groups, we utilized a comprehensive index called TO, which assesses surgical outcomes from multiple dimensions as a single desirable measure^38^. This widely used index has been extensively applied in abdominal surgery^39–41^. Recent studies have shown that achieving TO is associated with improved long-term prognosis in HCC patients undergoing hepatectomy^42,43^. TOLS, a derivative of TO specifically designed for liver surgery, was employed in our study. Our findings revealed no significant difference in the rate of achieving TOLS between the conversion therapy group and the surgery alone group, indicating that performing surgery after conversion therapy is both safe and technically feasible.

In the era of targeted and immunotherapy, clinicians often face a dilemma when treating patients with surgically resectable CNLC IIb/IIIa: direct surgery may not achieve optimal efficacy, while opting for conversion therapy as the initial approach could potentially delay surgical timing and forego operative opportunities. The findings of our study suggest that ‘conversion and resection’ represents a promising therapeutic approach in clinical settings for patients diagnosed with intermediate-advanced HCC who are initially considered eligible for surgical resection. However, our research is subject to certain limitations. Firstly, we utilized retrospective data from a single center with a restricted sample size. In future investigations, we aim to foster collaborative efforts in order to expand the sample size and consider designing randomized controlled prospective studies to mitigate potential selection bias. Secondly, the generalizability of our findings is constrained by variations in individual conversion therapy protocols and treatment durations. The primary reason for the heterogeneity of conversion protocols lies in the fact that conversion therapy has not yet become a standardized treatment for patients with surgically resectable CNLC IIb/IIIa HCC, leading to a lack of consensus on specific protocol selections. Future studies should further explore whether particular combinations of treatments enhance the prognosis of these patients.

## Conclusions

The implementation of conversion therapy has revolutionized the treatment approach for intermediate-advanced HCC. Our study suggests, in a preliminary manner, that surgical intervention following conversion therapy significantly reduces the incidence of postoperative MVI and prolongs RFS for patients with surgically resectable CNLC IIb/IIIa HCC. Furthermore, conversion therapy followed by hepatectomy represents a relatively safe treatment strategy. Investigating the underlying mechanisms of conversion therapy and identifying biomarkers to predict its survival benefits will facilitate future advancements in this therapeutic approach for intermediate-advanced HCC patients.

## Ethical approval

The study protocol received approval from the Ethics Committee of Sun Yat-sen Memorial Hospital, Sun Yat-sen University on 21 June 2023 (Approval No. SYSKY-2023-626-01).

## Consent

Considering that patients medical data was analyzed retrospectively, all informed consents were waived by the ethics committee. Of note, no patients-identifiable information was utilized.

## Sources of funding

This study was supported by the National Natural Science Foundation of China (No. 82103090); Medjaden Research Grants for Young Scientists (No. MJR20220907); Guangdong Basic and Applied Basic Research Foundation (No. 2021A1515012107, 2022A1515012391, 2023A1515010745).

## Author contribution

Z.F.M., Z.Y.X., J.L.Z., and K.M.: conceptualization, methodology, project administration, validation, and writing; Z.F.M., Z.Y.X., P.F.Y., K.W., W.D.W., Y.C.Y., Z.J.L., Z.L.L., H.K.W., J.L.Z., and K.M.: data curation and formal analysis; J.L.Z. and K.M.: supervision.

## Conflicts of interest disclosure

The authors declare that they have no conflicts of interest.

## Research registration unique identifying number (UIN)

The study was registered in the Chinese Clinical Trial Registry, registration number: ChiCTR2300077818.

## Guarantor

Dr Kai Mao had full access to all of the data in the study and takes responsibility for the integrity of the data and the accuracy of the data analysis.

## Data availability statement

The datasets used and analyzed during the current study are available from the corresponding author upon reasonable request.

## Provenance and peer review

Not commissioned, externally peer-reviewed.

## Presentation

None.

## Supplementary Material
