# Supplementary material for: Comparison of survival benefit and safety between surgery following conversion therapy versus surgery alone in patients with surgically resectable hepatocellular carcinoma at CNLC IIb/IIIa stage: a propensity score matching study
Source: Int J Surg. 2024 Feb 14;110(5):2910–21. doi: 10.1097/JS9.0000000000001193 (PMC11093426; doi:10.1097/JS9.0000000000001193)
Supplement: Supplementary file 2 [file js9-110-2910-s002.docx]

**Figure S1:** Forest plot of recurrence-free survival in subgroups of the entire cohort.


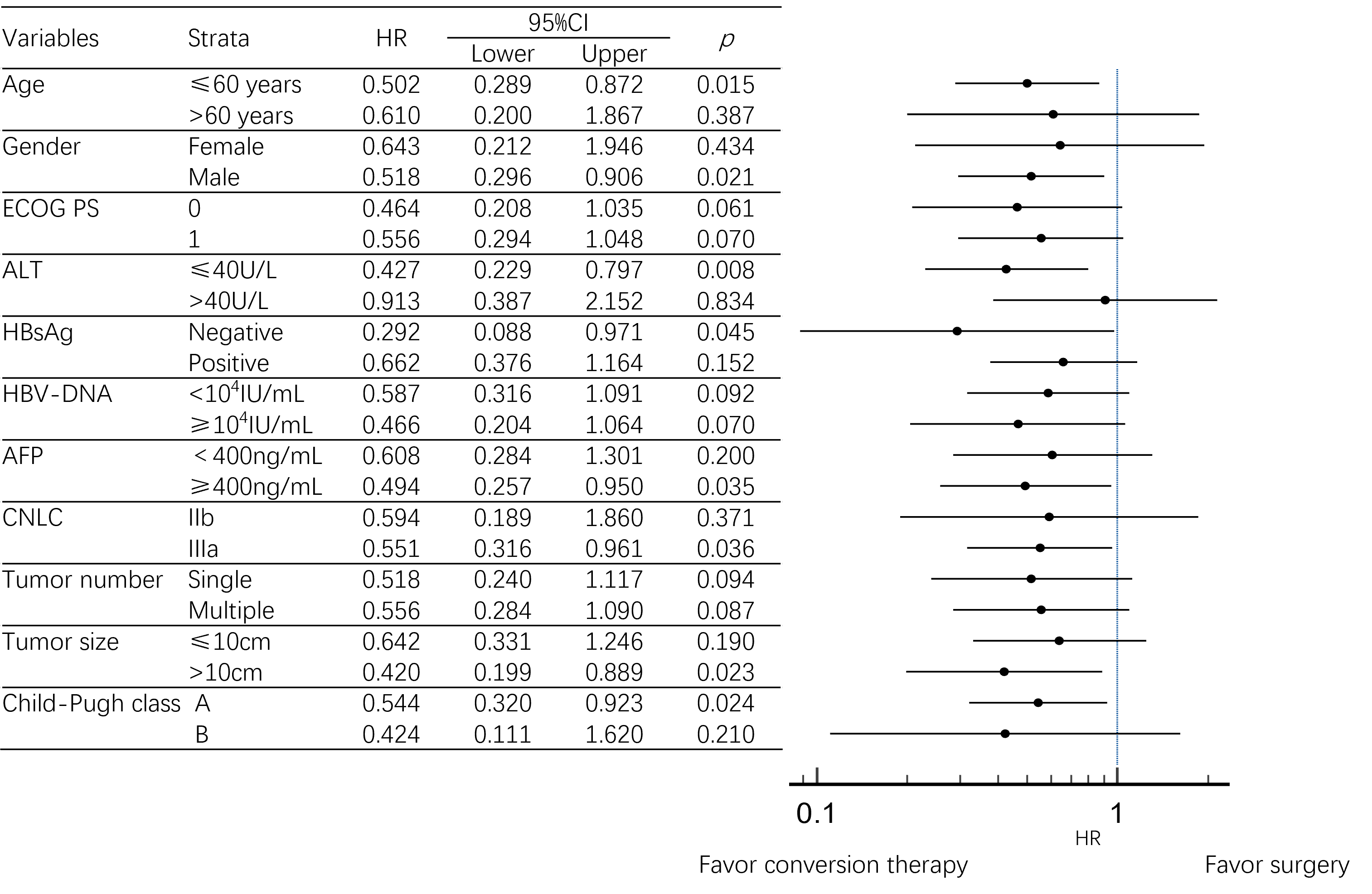


**Abbreviations:** ECOG PS: Eastern Cooperative Oncology Group Performance Status; ALT: alanine aminotransferase; HBsAg: hepatitis B surface antigen; AFP: alpha fetoprotein; CNLC: The China liver cancer staging system.

**Figure S2:** The potential mechanisms underlying the efficacy of conversion therapy in promoting enhanced patient survival.


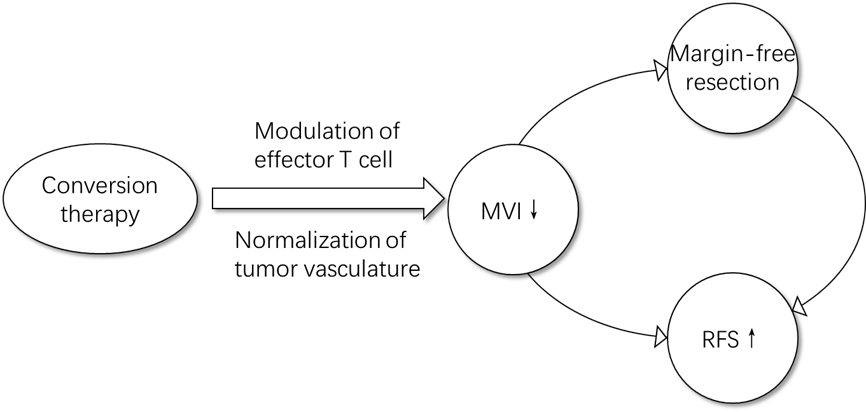


**Table S1:** Conversion therapy protocol and therapy cycle of patients in the conversion therapy group.

|  | **ICIs** | **therapy cycle** | **Antiangiogenic agents** | **therapy cycle** | **Local treatment** | **therapy cycle** |
| --- | --- | --- | --- | --- | --- | --- |
| 01 | Atezolizumab | 3 | Bevacizumab | 3 | HAIC | 3 |
| 02 | Atezolizumab | 4 | Bevacizumab | 4 | TACE | 2 |
| 03 | Camrelizumab | 5 | Apatinib |  | HAIC | 5 |
| 04 | Camrelizumab | 2 | Apatinib |  | HAIC | 2 |
| 05 | Camrelizumab | 4 | Apatinib |  | HAIC | 4 |
| 06 | Camrelizumab | 3 | Lenvatinib |  | HAIC | 3 |
| 07 | Camrelizumab | 4 | Lenvatinib |  | HAIC | 4 |
| 08 | Camrelizumab | 2 | Lenvatinib |  | HAIC | 2 |
| 09 | Camrelizumab | 1 | Apatinib |  | TACE | 1 |
| 10 | Camrelizumab | 5 | Lenvatinib |  | TACE | 2 |
| 11 | Pembrolizumab | 3 | Lenvatinib |  | HAIC | 3 |
| 12 | Pembrolizumab | 2 | Lenvatinib |  | HAIC | 2 |
| 13 | Pembrolizumab | 4 | Lenvatinib |  | HAIC | 4 |
| 14 | Pembrolizumab | 5 | Lenvatinib |  | HAIC | 5 |
| 15 | Pembrolizumab | 6 | Lenvatinib |  | HAIC | 6 |
| 16 | Pembrolizumab | 4 | Lenvatinib |  | HAIC | 4 |
| 17 | Tislelizumab | 6 | Lenvatinib |  | HAIC | 6 |
| 18 | Tislelizumab | 3 | Lenvatinib |  | HAIC | 3 |
| 19 | Tislelizumab | 5 | Lenvatinib |  | HAIC | 5 |
| 20 | Tislelizumab | 4 | Lenvatinib |  | HAIC | 4 |
| 21 | Sintilimab | 3 | Lenvatinib |  | HAIC | 3 |
| 22 | Sintilimab | 4 | Lenvatinib |  | HAIC | 4 |
| 23 | Sintilimab | 1 | Lenvatinib |  | TACE | 1 |
| 24 |  |  |  |  | HAIC | 11 |
| 25 |  |  |  |  | HAIC | 11 |
| 26 |  |  |  |  | HAIC | 2 |
| 27 |  |  |  |  | HAIC | 4 |
| 28 |  |  |  |  | TACE | 2 |
| 29 |  |  |  |  | TACE | 2 |
| 30 |  |  |  |  | TACE | 1 |

**Abbreviations:** ICIs: immune checkpoint inhibitors; HAIC: hepatic arterial infusion chemotherapy; TACE: transarterial chemoembolization.

**Table S2:** The dosages, administration frequency and administration routes of conversion therapy protocol.

|  | **Dosages** | **Administration frequency** | **Administration routes** |
| --- | --- | --- | --- |
| **ICIs** |  |  |  |
| Atezolizumab | 1200 mg | q3w | intravenous administration |
| Camrelizumab | 200 mg | q3w | intravenous administration |
| Pembrolizumab | 200 mg | q3w | intravenous administration |
| Tislelizumab | 200 mg | q3w | intravenous administration |
| Sintilimab | 200 mg | q3w | intravenous administration |
| **Antiangiogenic agents** |  |  |  |
| Bevacizumab | 15 mg/kg | q3w | intravenous administration |
| Apatinib | 250 mg | qd | oral administration |
| Lenvatinib | 8 mg for weight <60 kg 12 mg for weight ≥60 kg | qd | oral administration |
| **Local treatment** |  |  |  |
| HAIC^a^ |  |  |  |
| TACE^b^ |  |  |  |

^a^ HAIC treatment was divided into 3-week cycle. On day 1 in every cycle of treatment, the microcatheter was advanced into the hepatic artery and the drug was infused through the hepatic artery: oxaliplatin, 85 mg/m2 from hour 0-2 on day 1; levofolinate, 200 mg/m2 from hour 2-3 on day 1; and fluorouracil, 400 mg/m2 bolus at hour 3 on day 1 and 2,400 mg/m2 over 24 hours. After HAIC was completed, the catheter and sheath were removed.

^b^ For the TACE procedure, a catheter was inserted into the celiac trunk or superior mesenteric artery for arteriography. Then, a microcatheter was superselectively placed into the feeding arteries of the tumors. Chemolipiodolization was performed using 40 mg of epirubicin, 30 mg of lobaplatin and 750 mg of 5-fluorouracil mixed with lipiodol. Subsequently, embolization was performed with an absorbable gelatin sponge. Repeated TACE cycles were performed every 6 weeks.

**Abbreviations:** ICIs: immune checkpoint inhibitors; HAIC: hepatic arterial infusion chemotherapy; TACE: transarterial chemoembolization.

**Table S3:** Comparison of the postoperative complication between the conversion therapy group and surgery alone group.

| **Complication** | **Surgery alone** | **Conversion therapy** |
| --- | --- | --- |
| Pain | 18 | 14 |
| Fever | 10 | 6 |
| Vomiting | 7 | 9 |
| Hypokalemia | 8 | 6 |
| Ascites | 12 | 1 |
| Pleural effusion | 9 | 1 |
| Hypocalcemia | 5 | 5 |
| Hypertension | 6 | 3 |
| Bile leakage | 2 | 6 |
| Bleeding | 4 | 0 |
| Infection | 3 | 0 |
| Thrombocytopenia | 2 | 1 |
| Hyponatremia | 2 | 0 |
| Posthepatectomy liver failure | 1 | 0 |
| Hyperkalemia | 0 | 1 |
| Total | 89 | 53 |

**Table S4:** Tumor response and adverse events of conversion therapy.

| **Variables** | **n (%)** |
| --- | --- |
| **mRECIST** |  |
| CR | 5(16.7) |
| PR | 14(46.7) |
| SD | 5(16.7) |
| PD | 6(20.0) |
| **Pathological response** |  |
| pCR | 5(16.7) |
| MPR | 12(40.0) |
| **Adverse event** |  |
| Grade 1-2 | 64(90.1) |
| Grade 3 | 7(9.9) |
| Grade 4 | 0(0.0) |
| Grade 5 | 0(0.0) |

**Abbreviations:** mRECIST: modified response evaluation criteria in solid tumors; CR: complete response; PR: partial response; SD: stable disease; PD: progressive disease; pCR: pathological complete response; MPR: major pathological response.

**Table S5:** Adverse events experienced by patients during conversion therapy.

| **Adverse events** | **Any Grade(n，%)** | **Grade 1 or 2(n，%)** | **Grade 3(n，%)** |
| --- | --- | --- | --- |
| All adverse events | 28(90.3) | 27(87.1) | 7(22.6) |
| Anemia | 16(51.6) | 15(48.4) | 1(3.2) |
| AST elevation | 12(38.7) | 12(38.7) | 0(0.0) |
| ALT elevation | 7(22.6) | 7(22.6) | 0(0.0) |
| Thrombocytopenia | 7(22.6) | 7(22.6) | 0(0.0) |
| Rash | 6(19.4) | 3(9.7) | 3(9.7) |
| Hypertension | 6(19.4) | 6(19.4) | 0(0.0) |
| Hand-foot syndrome | 4(12.9) | 1(3.2) | 3(9.7) |
| Total bilirubin elevation | 4(12.9) | 4(12.9) | 0(0.0) |
| Neutropenia | 3(9.7) | 3(9.7) | 0(0.0) |
| Pruritus | 2(6.5) | 2(6.5) | 0(0.0) |
| Diarrhea | 1(3.2) | 1(3.2) | 0(0.0) |
| Constipation | 1(3.2) | 1(3.2) | 0(0.0) |
| Fatigue | 1(3.2) | 1(3.2) | 0(0.0) |
| Anorexia | 1(3.2) | 1(3.2) | 0(0.0) |
